# Morris Water Maze and Contextual Fear Conditioning Tasks to Evaluate Cognitive Functions Associated With Adult Hippocampal Neurogenesis

**DOI:** 10.3389/fnins.2021.782947

**Published:** 2022-01-03

**Authors:** Karina Hernández-Mercado, Angélica Zepeda

**Affiliations:** Departamento de Medicina Genómica y Toxicológia Ambiental, Instituto de Investigaciones Biomédicas, Universidad Nacional Autónoma de México, Ciudad de México, Mexico

**Keywords:** behavioral protocols, pattern separation, cognitive flexibility, hippocampal function, neurogenesis ablation, brain damage, functional recovery, plasticity

## Abstract

New neurons are continuously generated and functionally integrated into the dentate gyrus (DG) network during the adult lifespan of most mammals. The hippocampus is a crucial structure for spatial learning and memory, and the addition of new neurons into the DG circuitry of rodents seems to be a key element for these processes to occur. The Morris water maze (MWM) and contextual fear conditioning (CFC) are among the most commonly used hippocampus-dependent behavioral tasks to study episodic-like learning and memory in rodents. While the functional contribution of adult hippocampal neurogenesis (AHN) through these paradigms has been widely addressed, results have generated controversial findings. In this review, we analyze and discuss possible factors in the experimental methods that could explain the inconsistent results among AHN studies; moreover, we provide specific suggestions for the design of more sensitive protocols to assess AHN-mediated learning and memory functions.

## Introduction

The discovery of neurogenesis in the brain of adult mammals (Altman and Das, 1965; Kaplan and Hinds, 1977) contradicted the long-held dogma that the adult brain does not create new neurons. The idea was met with an intense skepticism that lasted over three decades. However, it is currently well accepted that new neurons are continuously generated in the subventricular zone of lateral ventricles and in the subgranular zone (SGZ) of dentate gyrus (DG) during the adult lifespan of a variety of mammals (Kuhn et al., 1996; Alvarez-Buylla and Lim, 2004; Pilz et al., 2018). The SGZ is a fine region between the hilus and the granular layer of the DG that harbors neural stem cells (NSCs) with different proliferative activity (Seri et al., 2001; Harris et al., 2021). In rodents, the neurogenic process in the DG comprises multiple steps that begin with the proliferation of NSCs. NSCs can either self-renew or give rise to intermediate progenitor cells, named type 2a cells, which have a high proliferative activity (von Bohlen und Halbach, 2007; Kempermann et al., 2015). These cells can self-renew or generate cells that further differentiate into so called type 2b cells, some of which still self-renew, while other progress into neuroblasts (Kempermann et al., 2004). During the first week after birth, neuroblasts migrate toward the inner zone of granule cell layer and exit from the cell-cycle becoming post-mitotic neurons (Brandt et al., 2003; Zhao, 2006). At this time point, about 60% of newborn neurons die by apoptosis, in a phase called early survival (Sierra et al., 2010). The new neurons that survive this phase extend their axons and form synapses with CA3 pyramidal neurons, while their dendrites grow toward the molecular layer and begin receiving inputs from neurons in the entorhinal cortex (Esposito, 2005; Zhao, 2006; Toni et al., 2008). Expression of NMDA receptors around 2–3 weeks after cell birth is important for the survival and network integration of the newly born neurons (Tashiro et al., 2006). Early synaptic integration of new neurons is followed by a phase of increased synaptic plasticity (around 4–6 weeks after birth) which may allow adult-born neurons to contribute to information processing in the DG (Wang et al., 2000; Schmidt-Hieber et al., 2004; Ge et al., 2007). In mice, new neurons reach structural and functional maturation at 6–8 weeks after birth (van Praag et al., 2002; Zhao, 2006), while the process occurs faster in rats (Snyder et al., 2009). The maturation stage as well as the time frame in which synaptic integration of newly born neurons occur are key factors regarding their contribution to DG network plasticity and cognition as they have unique physiological properties according to their developmental phase (Mongiat and Schinder, 2011).

The DG is a crucial structure that participates in hippocampus-dependent learning and memory (Hainmueller and Bartos, 2020). Thousands of new neurons are added to the DG of adult rats and mice daily (Cameron and McKay, 2001; Schauwecker, 2006). It has been shown that spatial learning modulates the rate of proliferation, survival, maturation and activation of newborn neurons (Gould et al., 1999; Kee et al., 2007; Tronel et al., 2010). Notably, spatial memory abilities in rodents have been positively correlated with the rate of adult hippocampal neurogenesis (AHN) (Drapeau et al., 2003; Berdugo-Vega et al., 2020). In contrast, the reduction of AHN impairs certain forms of hippocampal learning (Shors et al., 2002; Deng et al., 2009). While the specific contribution of AHN in cognition remains a central question, many studies have suggested that it promotes memory precision, resolution of conflicting information and memory consolidation (Aimone et al., 2011; Epp et al., 2016; Terranova et al., 2019; Berdugo-Vega et al., 2021).

The hippocampus is a key structure in navigation and in spatial learning and memory (Morris et al., 1982), while it is also essential for remembering aversive contexts (Phillips and LeDoux, 1992). Since the seminal work of O’Keefe (1976) a body of evidence has shown that cells in the hippocampus have a key role in the spatial representation of the environment. Thus, from the time that neurogenesis was fully accepted to occur in the adult hippocampus, researchers were interested in evaluating the functional contribution of new neurons to the hippocampal circuit as well as to hippocampus-dependent tasks.

Different behavioral paradigms have been used to study the functional implications of AHN in spatial learning and memory (for a review see, Lieberwirth et al., 2016). Morris water maze (MWM) and contextual fear conditioning and memory (CFC) are the most commonly used hippocampus-dependent tasks to evaluate episodic-like learning and memory (Phillips and LeDoux, 1992; Vorhees and Williams, 2006, 2014; Curzon et al., 2009). MWM is a reliable task that strongly correlates with hippocampal synaptic plasticity (Morris et al., 1986; Jeffery and Morris, 1993; Bannerman et al., 1995). From a practical point of view, it is easily adaptable to many experimental conditions that attempt to evaluate different aspects of cognition such as flexible and discriminative learning, as well as working and reference memory (Vorhees and Williams, 2006). As we will thoroughly revise in this work, MWM has shown that AHN is required for specific aspects of hippocampal-dependent cognition like memory precision (Berdugo-Vega et al., 2021), cognitive flexibility (Garthe et al., 2009), memory clearance (Epp et al., 2016), and long-term memory consolidation and reconsolidation (Snyder et al., 2005; Lods et al., 2021).

Contextual fear conditioning on the other hand, represents a form of associative learning widely used for studying episodic learning and memory as spatial representation is crucial for remembering an aversive context (Curzon et al., 2009). It has been extensively shown that the DG is key in supporting contextual fear memory (Phillips and LeDoux, 1992; Rudy et al., 2002; Sanders et al., 2003; Wiltgen, 2006; Bernier et al., 2017). Actually it has been observed that optogenetic stimulation of DG neurons which had been activated during fear acquisition drives fear expression (Liu et al., 2012; Ramirez et al., 2013; Redondo et al., 2014). As for the contribution of AHN to this task, it has been shown that newly born neurons promote contextual fear discrimination (Sahay et al., 2011), long-term memory consolidation (Kitamura et al., 2009; Kitamura and Inokuchi, 2014) and memory clearance (Gao et al., 2018). This task also allows investigating maladaptive fear responses such as excessive fear generalization, a hallmark of many anxiety and stress-related disorders, which have been shown to be modulated by AHN (Asok et al., 2019; for a review see, Besnard and Sahay, 2016).

Although the studies mentioned above have shown a causal relation between AHN and episodic-like memory through MWM and CFC paradigms, other studies have not (Raber et al., 2004; Meshi et al., 2006; Saxe et al., 2006; Clark et al., 2008; Zhang et al., 2008; Jaholkowski et al., 2009). Many factors could explain the discrepancy in the results. For instance, the diverse genetic background between rat and mice, as well as the diversity among the most common mice strains used for experimentation (C57BL/6, BALB/c, and 129Sv/J). In this regard, differences in the rate of adult hippocampal neuronal proliferation, survival rate, and maturation have been reported among strains (Kempermann et al., 1997; Snyder et al., 2009). Another possible factor contributing to the discrepancies is the experimental protocol used to evaluate performance in the absence of neurons: the amount of time elapsed between ablation and testing reflects the contribution to behavior of different cohorts of neurons at different stages of development (Gu et al., 2012). Also, the wide range of techniques to manipulate AHN including cytotoxic or chemical tools, irradiation, transgenic mice, and optogenetic modulation, may differ in efficacy, specificity, side effects, and the targeted newborn neuron cohort, therefore adding complexity to the interpretation of data (see Supplementary Tables 1, 2). Finally, it should also be considered that neurogenesis naturally declines with age in both, mice and rat (Kuhn et al., 1996) while its ablation may affect behavioral functions in an age-dependent manner (Martinez-Canabal et al., 2013) (see below).

Based on these factors, in this review, we will analyze and discuss the results in young-adult rodents that have provided evidence for the impact of AHN on spatial learning and memory using the MWM and CFC tasks. We also suggest some considerations to allow the design of more sensitive protocols for evaluating the role of AHN in such particular functions.

## The Morris Water Maze

The MWM is a behavioral paradigm that is used to evaluate spatial learning and memory in rodents and is commonly related to hippocampal synaptic plasticity (Morris et al., 1986; Vorhees and Williams, 2006). This test relies on the ability of rodents to navigate in a circular pool filled with water and find a safe place, which is a submerged hidden platform. The animals encode the platform location using distal visual cues surrounding the pool. Spatial learning is analyzed across repeated trials through which rodents learn the platform position (Vorhees and Williams, 2006). Spatial memory is usually evaluated through the time spent swimming over the goal quadrant when the platform is absent (D’Hooge and De Deyn, 2001).

In the field of adult neurogenesis, inconsistent results have been reported when this paradigm has been used. While some studies have found a positive effect of newly adult-generated neurons in learning and memory phases (Rola et al., 2004; Dupret et al., 2008; Farioli-Vecchioli et al., 2008; Zhang et al., 2008; Deng et al., 2009; Garthe et al., 2009), others have not (Shors et al., 2002; Madsen et al., 2003; Raber et al., 2004; Meshi et al., 2006; Saxe et al., 2006). Some possible factors that differ across the studies and that likely contribute to the discrepant results in each phase of this task are discussed below.

### Impact of Adult Neurogenesis on the Learning Phase of the Morris Water Maze Test

After suppressing adult neurogenesis, some studies reported alterations during the learning phase (i.e., animals had a higher latency or took a longer swim path to find the hidden platform) (Farioli-Vecchioli et al., 2008; Zhang et al., 2008; Garthe et al., 2009) while others did not (Shors et al., 2002; Madsen et al., 2003; Raber et al., 2004; Rola et al., 2004; Snyder et al., 2005; Meshi et al., 2006; Saxe et al., 2006; Deng et al., 2009; Jessberger et al., 2009; Martinez-Canabal et al., 2013). The reasons that alterations during the learning phase are reported only in some studies may be related to several factors including, but not limited to, (i) the accuracy of the parameters used to evaluate learning (see below allocentric vs. egocentric search strategies), (ii) the degree to which the behavioral protocol requires the animals to exhibit DG-associated behaviors, (iii) the saliency of visual cues located in the testing room, and (iv) the age of the animals at the time of AHN manipulation. We analyze such factors in the following paragraphs.

**FIGURE 1:**
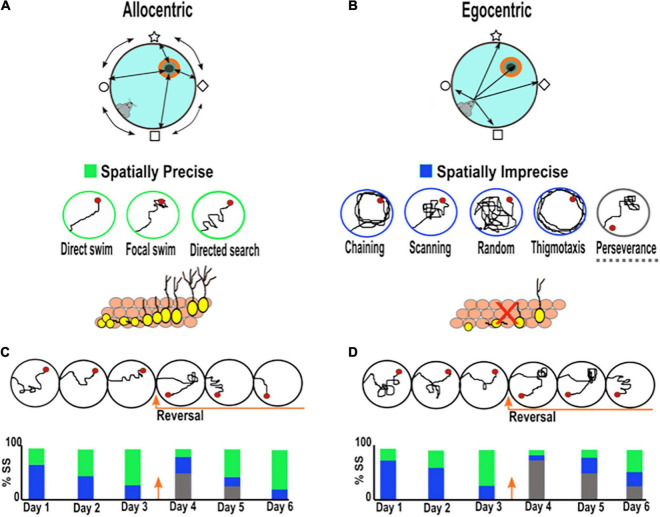
Searching strategies used by rodents during spatial learning in the Morris water maze. **(A)** Spatial allocentric strategy (object to object) is characterized by the ability to navigate using distal cues located outside the maze and at some distance from the rodent. The bottom panel shows representative examples of three allocentric search strategies listed from most precise to least precise (direct swim→ focal swim → directed search), which have been related to AHN. **(B)** Non-spatial egocentric strategy (self to object) characterized by the ability to navigate using internal cues independent of external cues based on a sequence of bodily movements. The bottom panel presents representative examples of six spatially imprecise egocentric search strategies (chaining, scanning, random, thigmotaxis, and perseverance). The decrease in AHN has been related to more imprecise search strategies. **(C,D)** Example of the search strategies adopted by rodents with normal **(C)** and low levels of neurogenesis **(D)**. The bottom bar charts illustrate the percentage of search strategies employed by rodents across training. Green bar: spatially precise search. Blue bar: spatially imprecise search. Gray bar: perseverance search. Rodents with low hippocampal neurogenesis require more training days before they begin to use spatially precise search strategies (green) than rodents with normal neurogenesis. When the platform changes to a new location, rodents with low neurogenesis spend more time searching over the old platform location (perseverance; gray bar) and adopt more imprecise search strategies (blue) than controls.

It is worth noting that during the learning phase of the MWM, rodents develop allocentric and egocentric search strategies to find the hidden platform (Vorhees and Williams, 2014; Rogers et al., 2017; Grech et al., 2018). When using allocentric strategies, the animals use the positional relationship among the distal cues around the pool to find the platform, independent of its self-position (world-centered view; object to object), while when utilizing egocentric navigation, the animal represents the platform location using internal cues relying on its self-position (animal-centered view; self-to-object) (Figures 1A,B; Rogers et al., 2017). Allocentric navigation is more dependent on the integrity and function of the hippocampus than egocentric navigation. The analysis of the search strategies developed by rodents during MWM learning reflects a suitable parameter for the evaluation of hippocampal spatial learning. Moreover, there is now a consensus that parameters such as the latency to find the platform or the path length are less reliable measurements for the evaluation of hippocampal spatial learning (Garthe et al., 2009; Gil-Mohapel et al., 2013; Rogers et al., 2017). The establishment of allocentric navigation strategies can be promoted by using different departure positions during the training phase in MWM. This requires that rodents learn and use the spatial relation among the distal cues in a flexible manner to successfully find the platform in each departure location.

The search strategy pattern adopted by the rodent can be classified according to the type of learning taking place from the least precise strategies (thigmotaxis, chaining, scanning, and random search) to the most precise (direct swim, focal search, and directed search) (Figures 1A,B; Garthe et al., 2009). Mice with normal levels of AHN begin the training phase using less precise search strategies (egocentric), but after a few trials, they develop highly precise search strategies (allocentric), such as a “focal or directed search,” to reach the platform (Figure 1C; Garthe et al., 2009). In contrast, mice with low levels of AHN need to perform more trials than rodents with normal levels to achieve allocentric searching patterns, and on the last day of training, animals scarcely conduct highly precise searches (Figure 1D; Garthe et al., 2009). In line with the latter, Gil-Mohapel et al. (2013) observed a positive correlation between the levels of AHN during aging and the type of search strategies, as young mice with high levels of AHN adopt more precise allocentric strategies, and old mice with lower neurogenesis levels exhibit less precise ones (Gil-Mohapel et al., 2013). Interestingly, it has been found that the use of precise hippocampal allocentric strategies during aging can be rescued by compensating for the age-related decline in AHN (Berdugo-Vega et al., 2020). In addition, it has been shown that increasing AHN in young mice with adequate spatial navigation strategies, promotes more spatially precise trajectories once hippocampal-navigation is acquired (Stone et al., 2011; Berdugo-Vega et al., 2021). These studies strongly suggest that AHN contributes to the development of precise allocentric search strategies during the learning phase of MWM, which points out a role of AHN in memory precision. Notably, unlike the search strategy pattern, some studies have shown that other parameters, such as latency and the swim distance to find the platform, remain relatively unaltered in mice with low neurogenesis during the learning period (see Supplementary Table 1; Garthe et al., 2009; Gil-Mohapel et al., 2013; Berdugo-Vega et al., 2020). Taken together, these studies suggest that the assessment of navigational learning strategies represents a key parameter to reveal important aspects of cognition associated with AHN function which do not become evident when assessing other classical parameters (i.e., latency and swim distance to reach the platform).

Regarding the degree of the hippocampal-dependency of the task, it has been proposed that MWM protocols promoting cognitive flexibility during the learning phase place a substantial demand on both hippocampal and AHN functions (Appleby and Wiskott, 2009; Burghardt et al., 2012). Pertinently, cognitive flexibility refers to the process by which previous knowledge or behaviors can be modified to adapt to new circumstances (Anacker and Hen, 2017). In rodents, cognitive flexibility can be evaluated using the reversal learning protocol in the MWM, where the animals have to learn a new platform location after having already learned one. The reversal learning protocol urges animals to form allocentric navigation strategies given that they have to learn the current positional relation between the distal visual cues around the pool and the new platform location (Anacker and Hen, 2017). After applying the reversal learning protocol, research groups have shown that rodents with reduced neurogenesis are unable to efficiently learn the new platform location (Dupret et al., 2008; Farioli-Vecchioli et al., 2008; Zhang et al., 2008; Garthe et al., 2009, 2016; for a discussion see Anacker and Hen, 2017; Abrous et al., 2021). In this case, rodents show prolonged times searching over the old platform location, a search pattern called perseverance. They also adopt more imprecise search strategies toward the new goal than rodents with normal levels of neurogenesis (Figures 1C,D and Supplementary Table 1). Remarkably, the increase of AHN reduces the perseverance search pattern after reversal learning (Berdugo-Vega et al., 2021). These results indicate that adult new neurons are needed to improve the integration of novel data when a main contingency has changed the previous consolidated information.

**FIGURE 2:**
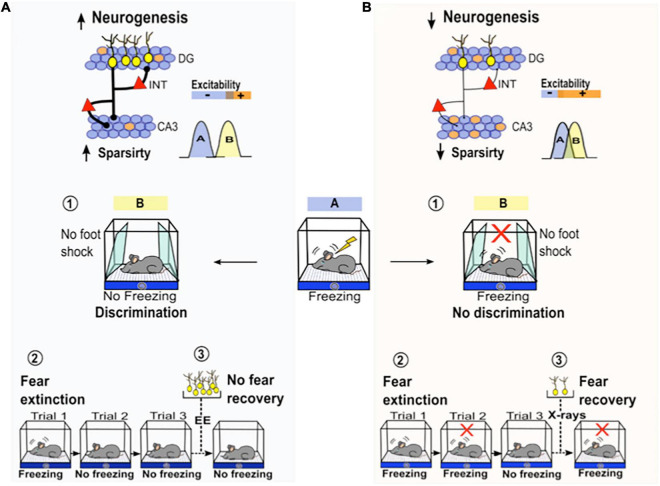
The roles of adult neurogenesis in different contextual fear-related behaviors. **(A)** Activation of adult born- neurons (yellow neurons) activates GABAergic inhibitory interneurons (red triangles) in the DG and CA3 (Restivo et al., 2015; Temprana et al., 2015), which may increase sparsity activity in the DG-CA3 circuit potentially promoting pattern separation. Left below, the function of pattern separation prevents interference among different neuron population codes that are active during two similar experiences: A (blue) vs. B (yellow). This process facilitates the ability of the animal to discriminate between the conditioned context (A, blue) and a similarly close safe context (sloping walls, B yellow, ➀). Through this mechanism, AHN may promote fear memory extinction by reducing the interference between an old aversive memory association (context with foot shock) and a new safe association (context with no foot shock, ➁). After fear memory extinction, the increase in AHN by environmental enrichment (EE) prevents spontaneous fear recovery ➂. **(B)** A decrease in adult born- neurons reduces sparsity activity in the DG-CA3 circuit which may impair pattern separation (Niibori et al., 2012; Denny et al., 2014). Bottom right, low neurogenesis impairs pattern separation, which decreases the ability of an animal to discriminate between closely similar contexts ➀. Impairment in pattern separation due to low neurogenesis may also impair fear memory extinction. Rodents with low neurogenesis are not able to reduce the conditioned fear response (freezing) as efficiently as mice with normal levels of neurogenesis (trial 2 vs. trial 3) during extinction training ➁. The reduction in AHN by irradiation (X-ray) promotes spontaneous fear recovery ➂.

A recent study reported that immature new neurons (DCX + without tertiary dendrites) are more likely implicated in tasks that demand cognitive flexibility than mature new neurons (Webler et al., 2019). Several lines of research indicate that immature new neurons may contribute to cognitive flexibility by enhancing the inhibitory tone in the DG-CA3 circuit to reduce the proactive interference between two similar experiences (i.e., old and new platform location) which is important to improve memory resolution (Kempermann, 2008; McAvoy et al., 2015; Besnard and Sahay, 2016; Berdugo-Vega et al., 2021). It has been suggested that newborn neurons can reduce the activity of mature granule cells through feedback inhibition (recruiting local interneurons) or direct inhibition via G protein-coupled inwardly rectifying potassium channels (Lacefield et al., 2012; Luna et al., 2019). Similarly, young neurons could inhibit CA3 pyramidal cells through feedforward inhibition (Rangel et al., 2013; Aimone, 2016). By modulating the timing of inhibition, adult-new neurons can increase the sparseness of two memory representations (Figures 2A,B).

Specifically, it has been shown that after reversal learning, mice with increased AHN display low neuronal activity in the suprapyramidal blade of the DG as well as in the CA3ab region. Thus, this sparsity decreases the overlap in memory representations from both structures (Berdugo-Vega et al., 2021). The contribution of immature new neurons to cognitive flexibility may also be explained by the “clearance hypothesis,” which states that the birth of neurons after learning induces the clearance of old hippocampus-dependent memories to allow new information to be stored (Josselyn and Frankland, 2012; Akers et al., 2014; Epp et al., 2016; Frankland and Josselyn, 2016). In fact, it has been observed that increasing AHN after initial learning facilitates the encoding of new conflicting learning (old vs. novel platform location) in MWM. Conversely, decreasing AHN after initial learning impedes the encoding of new conflicting learning (Epp et al., 2016). This indicates that AHN may reduce the proactive interference between two conflicting learning events by inducing the forgetting of the first one. It has been proposed that integration of new neurons into the hippocampal circuit modifies existing synaptic connections related to previous learning events by adding new connections (Weisz and Argibay, 2012). This structural remodeling of hippocampal circuits may change the pattern of activity associated to the previously learned event, which leads to memory decay over time and then allows the addition of new information (Frankland et al., 2013). Therefore, if AHN is ablated before learning, there are few chances that new neurons born afterwards incorporate into the hippocampal circuit. This may in turn prevent the clearance of previous learning and obstruct the addition of new information like learning a novel platform location. The role of AHN in cognitive flexibility has been thoroughly revised by Anacker and Hen (2017). Unveiling the precise mechanisms through which adult-born neurons promote cognitive flexibility is an issue worthy of further investigation. Still, these results highlight the importance of analyzing cognitive flexibility during MWM learning as a pertinent protocol to evaluate the contribution of AHN.

It has been shown that AHN is of particular importance in the performance of tasks that require spatial discrimination, where rodents have to distinguish between two highly similar contexts and learn that they are different from each other (McHugh et al., 2007; Clelland et al., 2009). In this regard, it has been reported that the posttraining ablation of newly born hippocampal neurons in mice impairs their ability to differentiate between two closely located similar cues in a water maze visual discrimination task (Arruda-Carvalho et al., 2011). This finding supports the specialized role of AHN in memory discrimination and agrees with the findings of other studies reporting that rodents with low neurogenesis have an impaired ability to correctly distinguish between two similar contexts in a fear conditioning task, or to recognize closely spaced arms in a radial maze (Clelland et al., 2009) and to distinguish objects in a two-choice touch screen spatial discrimination task (Clelland et al., 2009; Swan et al., 2014). The underlying mechanism associated to this discriminative behavior has not been well described. Still, as mentioned above, AHN may improve the discrimination of similar experiences by resolving the proactive interference between newly and previously learned spatial representations (Figures 2A,B). Moreover, computational models predict that AHN improves discrimination by temporal encoding of memories formed at different times through distinct population of young neurons (temporal separation) (Aimone et al., 2006). Likewise, AHN may promote discrimination by increasing memory resolution. The combination of immature with mature neurons as individual coding units, leads to a more precise and detailed codified memory. Mature neurons may strongly encode familiar features that have been previously experienced while immature neurons weakly encode novel features of the environment (Aimone et al., 2011). Memories incorporating more information (high resolution) will facilitate their discrimination. Therefore, the impact of new neurons is more evident when a MWM protocol requires the use of more DG-associated functions, such as reversal learning for cognitive flexibility, and spatial discrimination.

It has been shown that the saliency of the cues set around the testing room modulates the precision of the searching patterns in MWM task (Commins and Fey, 2019). Some features of the cues like shape, size, brightness, and distance from the pool or from the platform can contribute to its saliency. Rogers et al. (2017) observed that mice evaluated in an MWM paradigm using visual cues with high saliency showed higher percentage of spatial allocentric searching strategies than mice evaluated with low saliency visual cues. These observations denote that the saliency of the visual cues set around the testing area clearly influences the type of searching strategy (egocentric or allocentric) that is established. As mentioned above, several works have suggested that adult neurogenesis is required to establish precise allocentric search patterns during MWM learning (Dupret et al., 2008; Garthe et al., 2009; Stone et al., 2011; Gil-Mohapel et al., 2013; Berdugo-Vega et al., 2020). However, given that most neurogenesis studies do not describe the features of the cues employed around the testing room, it is unknown how the saliency of the cues could modulate (facilitate or hinder) the establishment of such precise allocentric searching patterns in rodents with low or increased AHN. Therefore, to provide a better understanding of the role of the saliency of visual cues located in the testing room, it is desirable that studies comprising neurogenesis behavioral evaluation clearly explain their features.

Finally, the age of animals at the time of AHN reduction is another factor that influences whether alterations in spatial learning are observed after neurogenesis manipulation. The generation of newborn neurons declines with the age of rodents. High rates of AHN are found in juvenile animals, moderate rates in adult, and low rates in old ages (Kuhn et al., 1996). In rats, an early age-related decrease in AHN is observed from 2 to 6 weeks and the decline persists until 48 weeks old (12 months old) when it reaches a steady rate (Heine et al., 2004). Likewise, in mice, a steep decline in AHN is observed from 4 to 8 weeks of age and then a progressive decline occurs until 72 weeks old (18 months old) (Bondolfi et al., 2004; Ben Abdallah et al., 2010; Gil-Mohapel et al., 2013). Notably, it has been shown that learning the location of a hidden platform in MWM is impaired only when neurogenesis is suppressed in juvenile mice (4–8 weeks old) but not in adult mice (8–12 weeks) or middle-age mice (44–48 weeks) (Martinez-Canabal et al., 2013). This finding is consistent with studies showing no effect of reducing AHN on MWM performances in adult rodents (see Supplementary Table 1; Shors et al., 2002; Madsen et al., 2003; Raber et al., 2004; Meshi et al., 2006; Saxe et al., 2006; Jaholkowski et al., 2009). In contrast, studies reducing neurogenesis in juvenile rodents (3–8 weeks old) have reported alterations in MWM performance (see Supplementary Table 1; Rola et al., 2004; Snyder et al., 2005; Dupret et al., 2008; Zhang et al., 2008; Deng et al., 2009; Garthe et al., 2009, 2016). Spatial learning alterations in younger rodents after neurogenesis suppression have been attributed to the fact that hippocampal circuitry has not yet fully developed (Bachevalier and Beauregard, 1993; Dumas, 2005). Once the hippocampus is completely mature, it might be less vulnerable to the reduction of AHN or such disruption could be compensated by pre-existing neurons (Martinez-Canabal et al., 2013). Therefore, the age of the animal at the time of AHN manipulation is another factor that should be considered for a proper design of behavioral experiments aimed at evaluating the role of AHN in hippocampal function.

Overall, the above mentioned works suggest that the decrease in AHN does not affect the general learning phase in the MWM but negatively impacts specific DG functions, such as the selection of precise allocentric spatial research strategies, cognitive flexibility, and spatial discrimination tasks. Moreover, given that the saliency of the visual cues located in the testing room can influence the use of allocentric searching patterns, the features of the visual cues must be carefully considered before the onset of the learning phase and should be described in detail. Finally, the age of rodents at the time of AHN reduction influences whether alterations in spatial learning are observed after neurogenesis manipulation. In the next section, we discuss results related to AHN and the memory phase in the MWM task.

### Impact of Adult Neurogenesis on Morris Water Maze Memory Performance

Regarding the evaluation of the role of adult-born neurons in memory using the MWM paradigm, there are several differences across studies that complicate their integration. To understand these results, we analyzed them according to the time elapsed between the end of the learning period and the beginning of the memory evaluation phase: short-term (≤24 h) and long-term (≥1 week) retention intervals.

#### Short-Term Memory

Several works have reported impairments in rodents with decreased AHN when memory retrieval was evaluated in retention time intervals ≤ 24 h. However, others have failed to find such impairments. Some possible explanations for the discrepancy among studies may be related, but not limited to the degree of the hippocampal dependency of the protocol employed (see above), the specificity of the methods used to modulate AHN, the age of the newborn neurons affected during testing and the possible stress generated when using intermediate probe tests.

In regard to the age of adult born neurons, it has been shown that immature new neurons ∼4–6 weeks old are particularly important for hippocampal memory function (Kee et al., 2007). New-born neurons at this age are more likely to be active than older neurons in mice that have been trained in the MWM (Kee et al., 2007). The absence of c-fos activation in new neurons ∼4–6 weeks old after MWM training is associated with an impairment in short-term memory recall (Farioli-Vecchioli et al., 2008). Interestingly, it has been observed that postlearning (but not prelearning) silencing or elimination of new neurons that are ∼4 weeks old impairs spatial memory expression in the MWM (Arruda-Carvalho et al., 2011; Gu et al., 2012). However, the postlearning silencing of adult-born neurons at ∼2 or ∼8 weeks old does not impair memory expression in MWM (Gu et al., 2012). Hence, the preferential recruitment of immature (∼4–6 weeks old) new neurons stresses their contribution to memory processing. These studies suggest that the impact of AHN on memory retrieval relies on a specific and small cohort of adult born neurons of a specific age. Furthermore, they also show that if immature new neurons (∼4–6 weeks old) are available at the time of learning, they come to form an essential component of hippocampal memory trace and then consistently influence short-term memory retrieval (Farioli-Vecchioli et al., 2008; Arruda-Carvalho et al., 2011; Gu et al., 2012; Epp et al., 2016). The specific manipulation of this small cohort of new neurons can explain why some studies have found alterations in memory retrieval while others have failed to do so. It has been observed that ~4–6 weeks old neurons express plastic properties (see below) that make them more likely to be recruited into spatial memory circuits compared to older (~8 weeks old) or younger (∼3 weeks old) granule cells. This however, does not rule out that younger or older cohorts of adult-born neurons may contribute to memory processing; still their functional activation during short-term memory retrieval seems to be limited.

Overall, as discussed in regard to the learning phase, the impact of AHN on memory formation seems to become more evident with protocol configurations that promote cognitive flexibility and spatial discrimination, while it is particularly important to consider the age of the new neurons when being tested.

#### Long-Term Memory

In addition to short-term memory findings, there are other studies involving the MWM that highlight the importance of new neurons in long-term memory. Notably, animals with reduced neurogenesis already before learning, spent less time swimming in the goal quadrant when memory was evaluated 1 or 4 weeks after the end of the learning phase (Snyder et al., 2005; Deng et al., 2009; Jessberger et al., 2009; Ben Abdallah et al., 2013). Conversely, the increase of AHN before learning has been associated with improvements in long-term memory retrieval (Wang et al., 2014). A recent work shows that adult-born neurons 1–2 and 6 weeks old at the time of learning are activated by long-term memory retrieval in MWM. Interestingly, the specific silencing of 1–2 week-old new neurons during long-term memory recall impairs remote memory reconsolidation. This suggests that immature new neurons that have been influenced by learning modulate the establishment of remote memories after activation (Lods et al., 2021).

Concerning long-term memory consolidation, it has been suggested that a given memory is initially dependent on the hippocampal region and that over time, cortical structures end up storing, at least partially, such memories (Wiltgen et al., 2004; Frankland and Bontempi, 2005; Preston and Eichenbaum, 2013; Kitamura et al., 2017). The mechanism by which hippocampal dependency decays over time and how the hippocampus and cortical regions interact to consolidate memories remains unknown. However, it has been shown that adult new neurons modulate the period during which a memory is hippocampus-dependent. The potential contributions of AHN to the mechanisms that underlie long-term memory consolidation have been hypothesized (Kitamura and Inokuchi, 2014; Terranova et al., 2019). Some of these proposals are further explained in the following section on contextual fear conditioning.

Considering all the above, we suggest some specifications that should be considered in the design of a sensitive protocol for the assessment of the functional relevance of AHN through the MWM: (1) precise parameters should be analyzed that reflect the formation of a spatial allocentric cognitive map to the escape platform, such as the searching strategies used by rodents during navigation; (2) cognitive flexibility and spatial discrimination behaviors should be promoted using the reversal learning and visual discrimination in the MWM task; (3) the saliency of the visual cues should be evaluated regarding whether they promote the establishment of hippocampal allocentric searching strategies; (4) the age of rodents at the time of AHN manipulation; and (5) the best time to manipulate AHN during the task should be identified.

## Contextual Fear Conditioning

Contextual fear conditioning is a behavioral procedure in which an animal learns to predict the presence of an aversive stimulus, like a foot-shock, through its association with a specific context (conditioning chamber). The foot-shock represents the unconditioned stimulus (US), and the context represents the conditioned stimulus (CS). The association between the US and the CS drives a classical fear response in rodents characterized by freezing (immobility) (Fanselow, 1980; Curzon et al., 2009; Maren et al., 2013). This behavioral paradigm highlights two phases: the learning or conditioning phase and memory evaluation (see below). Two of the major brain areas shown to be involved in contextual fear conditioning include the amygdala and the hippocampus. Studies attempting to identify the individual contribution of each structure to contextual fear conditioning have reported that lesions in the amygdala as well as in the hippocampus attenuate freezing during contextual retrieval, while lesions to the hippocampus interfere with contextual, but not with cued (i.e., sound) conditioning (Phillips and LeDoux, 1992; Zepeda et al., 2013).

Similar to studies in MWM, inconsistent results have been reported regarding the role of AHN in fear conditioning. However, a common factor among studies reporting alterations when AHN is decreased is that only contextual, but not cued fear memory is affected in rodents with low neurogenesis levels (see Supplementary Table 2). This highlights the fact that contextual fear representation, which is more dependent on hippocampal function, is indeed modulated by AHN.

Below, we discuss some factors that differ across the studies and that likely contribute to the discrepant results. Moreover, we briefly analyze other fear-related behaviors that have been associated with the function of AHN, such as fear extinction, spontaneous fear recovery, and fear generalization.

### Adult Neurogenesis in the Conditioning Phase

During the conditioning phase, animals learn to associate a foot shock with a specific context. Rodents are placed into the context (conditioning chamber), and then single or multiple foot shocks are delivered. Learning performance is evaluated through the progressive increase in a freezing response across the training session (only for multiple training, not for single training sessions). The methodology employed during conditioning varies considerably among AHN studies. These variations may yield discrepancies among the findings. Here, we highlight four factors related to the conditioning protocol that may impact CFC results.

#### First: Training Severity

This refers to the intensity, duration and the number of foot shocks delivered to rodents during the conditioning phase; these factors determine the time of incubation and the brain areas involved in processing fear-associated memories (Poulos et al., 2016). Training severity is quite variable among adult neurogenesis studies; some use intense training methods, such as multiple foot shocks (Winocur et al., 2006; Kitamura et al., 2009; Snyder et al., 2009), while others employ mild training methods such as a single foot shock (Imayoshi et al., 2008; Pan et al., 2012). Notably, memory is more consistently affected after a single foot shock than after multiple foot shocks in rodents with diminished AHN (Saxe et al., 2006; Winocur et al., 2006; Zhang et al., 2008; Snyder et al., 2009; Pan et al., 2012). It has been considered that multiple foot shocks produce greater emotional arousal than a single foot shock in rodents (Wiltgen, 2006; Drew et al., 2010; Poulos et al., 2016). Pertinently, greater arousal could strengthen contextual fear acquisition in extrahippocampal sources (neocortex) and then mask the participation of the hippocampus and adult neurogenesis (Wiltgen, 2006; Drew et al., 2010). The fact that a single shock is particularly sensitive to AHN has led to the hypothesis that adult-born new neurons are specifically important for encoding mild events into memory (low emotional arousal; single shock) rather than for encoding intense events into memory (high emotional arousal; multiple foot shock). Therefore, a single trial seems to be a more suitable training method for the evaluation of the role of AHN in CFC.

#### Second: Rodent Nociception

The sensory perception of foot shocks in rodents can modulate their fear response during memory evaluation. Moreover, the sensory perception of a foot shock can result in a decreased conditioned fear or even the lack of a fear response (i.e., non-conditioned animal) if the shock is perceived as a low-intensity stimulus. In contrast, the sensory perception of a foot shock can increase animals’ conditioned fear or even result in a maladaptive fear response (i.e., fear generalization) if the shock is perceived as a high-intensity stimulus (Baldi, 2004). Thus, the impact of a foot shock can be differently perceived in rodents previously exposed to aversive manipulation and can be differently perceived among different mouse strains (genetic background) (Wotjak, 2019). In adult neurogenesis studies, rodents are often subjected to invasive procedures, such as ablation or tracing of new neurons prior to the commencement of behavioral protocols (i.e., surgeries or multiple drug injections) (Saxe et al., 2006; Deng et al., 2009; Arruda-Carvalho et al., 2011; Gu et al., 2012; Huckleberry et al., 2018). These procedures may represent aversive experiences that affect the sensory perception of the rodent to a further negative event, such as a foot shock. Moreover, some adult neurogenesis studies use a mixed genetic background of mice among their experimental groups (transgenic vs. wild-type mice) (Saxe et al., 2006; Deng et al., 2009; Denny et al., 2012), which likely increases the variability in sensory perception and alters the freezing response. To exclude the “sensory perception” variable in their results, some authors have employed two ablating methods (irradiation and transgenic mice) (Saxe et al., 2006), while others have evaluated the outcomes of a shock sensitivity curve test before conducting the behavioral test (Tronel et al., 2012). Therefore, the impact of shock intensity in a cohort of independent animals should be evaluated and adjusted before behavioral procedures begin. These results should also be reported to enhance the comparability of data among different AHN studies.

#### Third: The Preshock Time Interval

This refers to the interval between the introduction of the rodent into the context and the delivery of the first foot shock. The preshock time is important for context encoding and memory resolution, as animals need time to form a representation of the context before the shock is presented (Drew et al., 2010). If a shock is delivered immediately after the introduction of the rodent in the context, without a preshock interval, contextual learning does not occur. In particular, AHN seems to be important for memory resolution when the preshock interval is short (Drew et al., 2010; Denny et al., 2012). Mice with reduced AHN exhibit impairments in contextual fear memory only when a single shock is delivered after a preshock period of 180 s but not when it is prolonged to 360 s (Drew et al., 2010; Denny et al., 2012). These studies suggest that new neurons are necessary for the rapid acquisition of context memories, a role that has also been attributed to the DG-CA3 circuit (Nakashiba et al., 2012; Hainmueller and Bartos, 2020). Therefore, a preshock interval of 180 s seems to be a suitable parameter in the evaluation of the implications of adult neurogenesis in CFC.

#### Fourth: The Context Configuration

The DG is critical for associating individual items with their context, which comprises the background setting of a spatial environment (Lee and Jung, 2017). The features of the conditioning chamber (context) vary extensively among adult neurogenesis studies. Some groups use chambers that are insulated inside a cabinet, which reduces visual access to the cues surrounding the chamber (Warner-Schmidt et al., 2008; Drew et al., 2010; Denny et al., 2012). In contrast, others employ non-insulated chambers with clear walls, which increase access to visual cues (Winocur et al., 2006; Farioli-Vecchioli et al., 2008; Imayoshi et al., 2008; Snyder et al., 2009; Pan et al., 2012). Given that these studies use different conditioning parameters, it is difficult to determine to what extent access to visual cues around the environment may influence animals’ behavioral results. Hence, it would be desirable to establish whether mice with low neurogenesis need longer preshock intervals to encode a complex context (non-insulated chamber with visual access to the environment) than a simple one (insulated chamber with no visual access to the environment). Further experiments are necessary to evaluate the adequate combination of these variables (severity of training, preshock time, and context configuration) to design an optimal conditioning protocol in AHN studies.

Overall, the impact of AHN seems to become more evident when the conditioning phase consists of mild training (single foot shock) and a short preshock interval (180 s). Moreover, the reactivity of the rodent to a foot shock and access to visual cues in the conditioning context are variables that should be considered in the interpretation of the results.

### Impact of Adult Neurogenesis in Contextual Fear Memory

During the memory phase, rodents are placed in the context again but do not receive any foot shocks. If they remember the context as an aversive experience, the animals display freezing behavior (immobility). Contextual memory is evaluated through the percentage of time that rodents freeze during memory assessment. While a high percentage of freezing reflects adequate contextual fear memory establishment, lower levels of freezing indicate degraded or impaired contextual fear memory. Interestingly, in the AHN literature, there are several studies showing that newly generated adult neurons are necessary for optimal contextual fear memory function (Saxe et al., 2006; Winocur et al., 2006; Farioli-Vecchioli et al., 2008; Imayoshi et al., 2008; Hernández-Rabaza et al., 2009; Snyder et al., 2009; Drew et al., 2010; Gu et al., 2012; Pan et al., 2012; Huckleberry et al., 2018). Here, we briefly describe two characteristics of AHN that are important for the resolution of contextual fear memory. Notably, these traits have also been observed through the MWM paradigm, which indicates their robust impact on hippocampal memory (see the MWM section).

**FIGURE 3:**
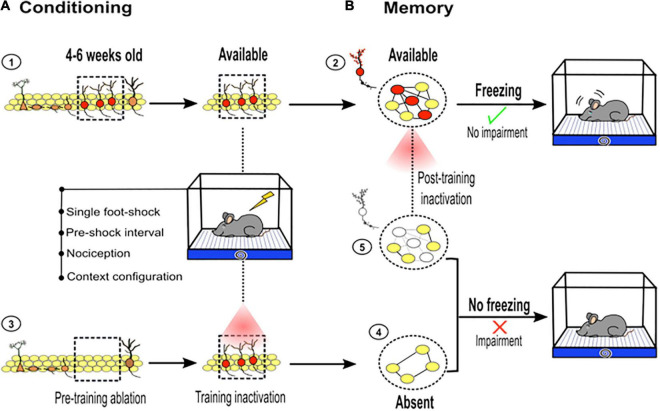
Adult hippocampal neurogenesis and contextual fear learning and memory. **(A)** ➀ Immature new neurons between 4 and 6 weeks old (red, inside dotted square) are particularly important for hippocampal memory function. Immature new neurons available at the time of the training become a part of the memory trace: ➁ dotted circle, young neurons (red) connected (black lines) with mature neurons (yellow). However, if they are absent before training or optogenetically silenced during conditioning ➂, their contribution to the memory trace is restricted ➃. **(B)** As part of the memory trace, young neurons contribute to an adequate memory retrieval, which is reflected by freezing behavior. Pretraining ablation or silencing of new neurons during conditioning, impairs memory retrieval, which is reflected by diminished freezing behavior. If immature new neurons are silenced during retrieval ➄, memory impairment is observed (diminished freezing). This highlights the role of immature new neurons between 4 and 6 weeks old as a critical component of memory traces.

First, immature new neurons 4–6 weeks old are particularly necessary for contextual fear memory (Farioli-Vecchioli et al., 2008; Denny et al., 2012; Gu et al., 2012; Kirby et al., 2012). The specific loss or silencing of this cohort of new neurons degrades contextual fear memory retrieval (Figure 3A; Gu et al., 2012; Huckleberry et al., 2018). It has been suggested that immature neurons become an essential substrate of memory because they temporally express plastic properties, such as a decreased long-term potentiation (LTP) induction threshold, an expression of the NR2B subunit of the *N*-Methyl-D-aspartate (NMDA) receptor and type T-Ca^2++^ channels (Schmidt-Hieber et al., 2004; Ge et al., 2007; Gu et al., 2012). In addition, these immature neurons form stable and functional synapses with CA3 pyramidal neurons at this age, which is an important event for hippocampal cognition (Toni et al., 2007, 2008; Gu et al., 2012). These temporally plastic proprieties enhance the plasticity of these neurons and may increase the probability of them being incorporated into a memory engram. As a component of a memory engram, immature neurons likely influence hippocampal short-term memory recall.

Second, postlearning manipulation (ablating or silencing) of these immature neurons (∼4–6 weeks old) has a more disruptive effect on memory resolution than prelearning manipulation (Gu et al., 2012). This suggests that the impact of immature neurons in short-term memory is more evident if the neurons are available during learning, because they come to form an enduring component of hippocampal memory trace (Figures 3A,B). However, their influence on short-term memory is less evident or less obvious if the neurons are absent during learning, which may limit their incorporation into the memory trace. Nevertheless, as extensively discussed above, there are some methodological features used during the learning phase that help highlight the role of adult neurogenesis in memory. Taken together, using pre- or postlearning manipulation of adult neurogenesis, studies have pointed out the importance of this small and temporal cohort of new neurons in hippocampal memory function.

#### Long-Term Memory

In agreement with MWM results, there are studies using contextual fear memory that support the role of adult neurogenesis in long-term memory (Winocur et al., 2006; Arruda-Carvalho et al., 2011). These studies suggest that AHN modulates the transfer of memories from the hippocampus to cortical structures for long-term storage by shortening the time dependence of memories in the hippocampus. It has been shown that decreased levels of adult neurogenesis prolong the time dependence of contextual fear memories in the hippocampus, while increased levels shorten this time dependence (Kitamura et al., 2009). Moreover, it has been observed that the continuous addition of adult-born new neurons regulates the capacity of the hippocampus to store new memories, preventing saturation of the circuit. A decrease in neurogenesis by irradiation delays memory capacity recovery by prolonging LTP persistence. In contrast, an increase in neurogenesis by running accelerates memory capacity recovery by shortening LTP persistence (Alam et al., 2018). These results are in agreement with studies indicating that adult neurogenesis increases the network capacity for new information, promoting the removal of old information (Feng et al., 2001; Aimone et al., 2009; Frankland and Josselyn, 2016). By shortening the time dependence of a memory in the hippocampus and increasing the capacity to store new memories in the hippocampus, adult neurogenesis may promote memory formation (Terranova et al., 2019). Although, the precise mechanism through which AHN promotes long-term memory storage is still unknown, some possible implications have been speculated (for review see Terranova et al., 2019).

Recent studies have stressed the physiological relevance of the activity of memory-engram cells in memory consolidation through the CFC task (Kitamura et al., 2017; Tonegawa et al., 2018). Memory-engram cells related to contextual fear memory in the DG and the medial prefrontal cortex are associated with short- and long-term information storage, respectively. Memory engram cells in the DG are active early after the learning period and become inactive over time. Conversely, memory-engram cells from the medial prefrontal cortex are inactive early after the learning period and become active over time (for a discussion see Tonegawa et al., 2018). It is currently unknown how memory-engram cells become active or inactive over time; however, it is likely that new neurons can modulate these states. The continuous integration of newly generated neurons into the hippocampal circuit can disrupt the already established synaptic connections among memory engram cells (Kitamura and Inokuchi, 2014; Tonegawa et al., 2018; Terranova et al., 2019). This mechanism may in turn inactivate the memory engram in the DG over time. New technological tools that combine transgenic mice and optogenetic engineering to identify and modulate the activity of memory engram cells generated during contextual fear memory formation could help clarify the specific roles of adult neurogenesis in memory processes.

### The Role of Adult Neurogenesis in Other Contextual Fear-Related Behaviors

After establishing contextual fear conditioning, it is possible to analyze other DG-mediated cognitive behaviors, such as fear extinction (Ji and Maren, 2007), fear recovery (Martínez-Canabal et al., 2019), and fear generalization (Asok et al., 2019). Next, we briefly describe the role of adult neurogenesis in the abovementioned contextual fear-related behaviors.

#### Contextual Fear Memory Extinction

In this process, extinction is defined as a decline in the conditioned fear response following repeated exposure to the context in the absence of foot shock (Ji and Maren, 2007). After extinction, the rodent learns that the context is no longer a predictor of the aversive shock (Ji and Maren, 2007). In the field of adult neurogenesis, studies have shown that mice with low levels of neurogenesis are not able to diminish the conditioned fear response as efficiently as mice with normal levels of neurogenesis during extinction training (Deng et al., 2009; Pan et al., 2012, 2013). Still, AHN may participate in fear memory extinction by reducing the overlap between both memory associations (Figure 2A; Kempermann, 2008; McAvoy et al., 2015; Besnard and Sahay, 2016).

#### Spontaneous Contextual Fear Recovery

After fear extinction, the expression of fear often returns as time passes, a phenomenon known as spontaneous fear recovery (Ji and Maren, 2007). It has been suggested that during recovery of an extinguished fear, the memory trace related to the fear response (spontaneous fear recovery) competes with the memory trace related to the lack of a fear response (extinguished fear) for the expression of the related behavioral response (Ji and Maren, 2007). Martínez-Canabal et al. (2019) investigated how AHN affects spontaneous fear recovery and its underlying brain network activity. The authors found that an increase in adult neurogenesis after but not before extinction prevented spontaneous fear recovery. In contrast, the reduction in adult neurogenesis after but not before extinction promoted spontaneous fear recovery (Figures 2A,B). Further experiments are necessary to develop a better understanding of the role of new neurons in spontaneous fear recovery. However, this study adds information to previous results in two ways. First, the posttraining modulation of adult neurogenesis (after extinction but not before) was shown to be more disruptive because new neurons are already committed to a memory trace (Arruda-Carvalho et al., 2011) and second, adult neurogenesis may contribute by reducing the proactive interference between both fear memory responses (Kempermann, 2008; McAvoy et al., 2015; Besnard and Sahay, 2016).

#### Fear Generalization

After associating the aversive presence of a foot shock to a specific context, rodents can show fear responses even in a similar innocuous context, which reflects their inability to make fine spatial discriminations (Lissek et al., 2010; Asok et al., 2019). The uncontrollable expression of fear in a safe context is known as fear generalization (Lissek et al., 2010; Asok et al., 2019). Indeed, rodents with low levels of neurogenesis in the DG have a reduced ability to discriminate between aversive and similar safe contexts (Nakashiba et al., 2012; Tronel et al., 2012). In contrast, rodents with high levels of neurogenesis increase their ability to discriminate between both contexts (Sahay et al., 2011). Several studies suggest that adult neurogenesis in the DG facilitates spatial discrimination and limits fear generalization by promoting pattern separation in the DG-CA3 circuit (Restivo et al., 2015; Temprana et al., 2015; Drew et al., 2016; Figures 2A,B). Adult neurogenesis facilitates the discrimination of similar contexts by regulating cell activity in the DG and CA3 and by preventing an overlap in the population code; the “population code” refers to the group of cells that are active during the encoding of a given context (Burghardt et al., 2012; Niibori et al., 2012). Several studies suggest that adult neurogenesis in the DG reduces the overlap of population code by activating inhibitory GABAergic neurons that lead to sparse cell activity in the DG and in CA3 (Burghardt et al., 2012; Niibori et al., 2012; Restivo et al., 2015; Temprana et al., 2015; Besnard and Sahay, 2016; Drew et al., 2016; Drew and Huckleberry, 2017; Figure 2A).

In summary, there is high heterogeneity among the protocols used to evaluate the function of neurogenesis through contextual fear. We posit that there are four possible conditioning parameters that may influence behavioral results and that experimenters should carefully consider in their experimental design: (1) the severity of the trial (multiple vs. single shock), (2) the preshock time interval, (3) the context configuration and, (4) the impact of the side effects generated by the ablating methods on mouse nociception. Finally, through pattern separation or by the forgetting of a previously learned memory, AHN may modulate other fear-related cognitive behaviors, such as fear extinction, fear recovery, and fear generalization.

## Conclusion

Adult neurogenesis occurring in the rodent hippocampus has been widely accepted for a few decades now. Efforts to understand the role of adult-born neurons in behavior started almost hand-in-hand with these observations. The refinement of strategies to analyze, target and manipulate adult-born cells has provided clear evidence of the role of neurogenesis in behavior. Moreover, the dissection of the elements involved in the design of behavioral tasks has shown to be crucial for understanding the subtle roles of new neurons in episodic-like learning and memory. The consideration of each of the discussed elements for the implementation of a task when addressing the role of neurogenesis in behavior may provide a refined framework for discussing the obtained results.

## Author Contributions

KH-M and AZ wrote the manuscript. KH-M prepared the figures. AZ revised the manuscript. Both authors contributed to the article and approved the submitted version

## Conflict of Interest

The authors declare that the research was conducted in the absence of any commercial or financial relationships that could be construed as a potential conflict of interest.

## Publisher’s Note

All claims expressed in this article are solely those of the authors and do not necessarily represent those of their affiliated organizations, or those of the publisher, the editors and the reviewers. Any product that may be evaluated in this article, or claim that may be made by its manufacturer, is not guaranteed or endorsed by the publisher.
